# Preclinical pharmacokinetics, biodistribution, radiation dosimetry and toxicity studies required for regulatory approval of a phase I clinical trial with ^111^In-CP04 in medullary thyroid carcinoma patients

**DOI:** 10.1016/j.ejps.2016.05.011

**Published:** 2016-05-14

**Authors:** Theodosia Maina, Mark W. Konijnenberg, Petra KolencPeitl, Piotr Garnuszek, Berthold A. Nock, Aikaterini Kaloudi, Marko Kroselj, Katja Zaletel, Helmut Maecke, Rosalba Mansi, Paola Erba, Elisabeth von Guggenberg, Alicja Hubalewska-Dydejczyk, Renata Mikolajczak, Clemens Decristoforo

**Affiliations:** a Molecular Radiopharmacy, INRASTES, NCSR “Demokritos”, Athens, Greece; b Department of Radiology and Nuclear Medicine, Erasmus MC, Rotterdam, The Netherlands; c Department of Nuclear Medicine, University Medical Centre Ljubljana, Slovenia; d Radioisotope Centre POLATOM, National Centre for Nuclear Research, Otwock, Poland; e Department of Nuclear Medicine, University Hospital Freiburg, Germany; f Department of Nuclear Medicine, Azienda Ospedaliero-Universitaria Pisana, Pisa, Italy; g Department of Nuclear Medicine, Innsbruck Medical University, Austria; h Department of Endocrinology, Jagiellonian University Medical College, Krakow, Poland

**Keywords:** Radiopharmaceutical development, Medullary thyroid cancer, CCK2R-targeting of tumours, Gastrin-radioligand, Radionuclide therapy, Clinical translation

## Abstract

**Introduction:**

From a series of radiolabelled cholecystokinin (CCK) and gastrin analogues, ^111^In-CP04 (^111^In-DOTA-(DGlu)_6_-Ala-Tyr-Gly-Trp-Met-Asp-Phe-NH_2_) was selected for further translation as a diagnostic radiopharmaceutical towards a first-in-man study in patients with medullary thyroid carcinoma (MTC). A freeze-dried kit formulation for multicentre application has been developed. We herein report on biosafety, in vivo stability, biodistribution and dosimetry aspects of ^111^In-CP04 in animal models, essential for the regulatory approval of the clinical trial.

**Materials and methods:**

Acute and extended single dose toxicity of CP04 was tested in rodents, while the in vivo stability of ^111^In-CP04 was assessed by HPLC analysis of mouse blood samples. The biodistribution of ^111^In-CP04 prepared from a freeze-dried kit was studied in SCID mice bearing double A431-CCK2R(±) xenografts at 1, 4 and 24 h pi. Further 4-h animal groups were either additionally treated with the plasma expander gelofusine or injected with ^111^In-CP04 prepared by wet-labelling. Pharmacokinetics in healthy mice included the 30 min, 1, 4, 24, 48 and 72 h time points pi. Dosimetric calculations were based on extrapolation of mice data to humans adopting two scaling models.

**Results:**

CP04 was well-tolerated by both mice and rats, with an LD_50_ > 178.5 μg/kg body weight for mice and a NOAEL (no-observed-adverse-effect-level) of 89 μg/kg body weight for rats. After labelling, ^111^In-CP04 remained >70% intact in peripheral mouse blood at 5 min pi. The uptake of ^111^In-CP04 prepared from the freeze-dried kit and by wet-labelling were comparable in the A431-CCK2R(+)-xenografts (9.24 ± 1.35%ID/g and 8.49 ± 0.39%ID/g, respectively; *P* > 0.05). Gelofusine-treated mice exhibited significantly reduced kidneys values (1.69 ± 0.15%ID/g vs. 5.55 ± 0.94%ID/g in controls, *P* < 0.001). Dosimetry data revealed very comparable effective tumour doses for the two scaling models applied, of 0.045 and 0.044 mSv/MBq.

**Conclusion:**

The present study has provided convincing toxicology, biodistribution and dosimetry data for prompt implementation of the freeze-dried kit formulation without or with gelofusine administration in a multicentre clinical trial in MTC patients.

## 1 Introduction

Medullary thyroid cancer (MTC) is a neuroendocrine tumour of the parafollicular or C cells of the thyroid, not accumulating iodine. It accounts for 3% to 5% of thyroid carcinomas (Pacini et al., 2010; Wells et al., 2015). In many cases disease has already metastasized at the time of diagnosis with limited therapeutic options. Long-term responses by radiotherapy or systemic therapy are uncommon (Pacini et al., 2010) and new targeted chemotherapy approaches (like tyrosine kinase inhibitors, TKI) show significant toxicity and do not change overall survival is (Nikiforova and Nikiforov, 2008; Zhang et al., 2009). MTC therefore still remains a highly challenging cancer for both the patient and the physician.

Molecular nuclear medicine can play an important role in diagnosis (SPECT/CT, PET/CT) and therapy of advanced MTC (Reubi and Maecke, 2008). Molecular imaging and peptide receptor radionuclide therapy (PRRT) approaches can exploit the overexpression of cholecystokinin subtype 2 receptors (CCK2R) at an incidence of over 90% in MTC (Reubi et al., 1997; Reubi and Waser, 1996). This finding has motivated a number of research groups to develop site-specific radiolabeled analogues of cholecystokinin-8 (CCK-8; Asp-Tyr-Met-Gly-Trp-Met-Asp-Phe-NH_2_) or minigastrin (MG, Leu-(Glu)_5_-Ala-Tyr-Gly-Trp-Met-Asp-Phe-NH_2_). (Behr and Behe, 2002; Froberg et al., 2009; Nock et al., 2005; Sosabowski et al., 2007; von Guggenberg et al., 2004; von Guggenberg et al., 2007).

The very high kidney retention of these tracers, which limits their application for radionuclide therapy (Behe and Behr, 2002), has stimulated further research which revealed the importance of the multi-negative charges of the penta-Glu chain but also of the secondary peptide structure on the in vivo profile of resulting minigastrin radioligands (Breeman et al., 2008; Good et al., 2008; Kolenc-Peitl et al., 2011; Kolenc Peitl et al., 2015; Mather et al., 2007; von Guggenberg et al., 2009, 2012).

**Fig. 1:**
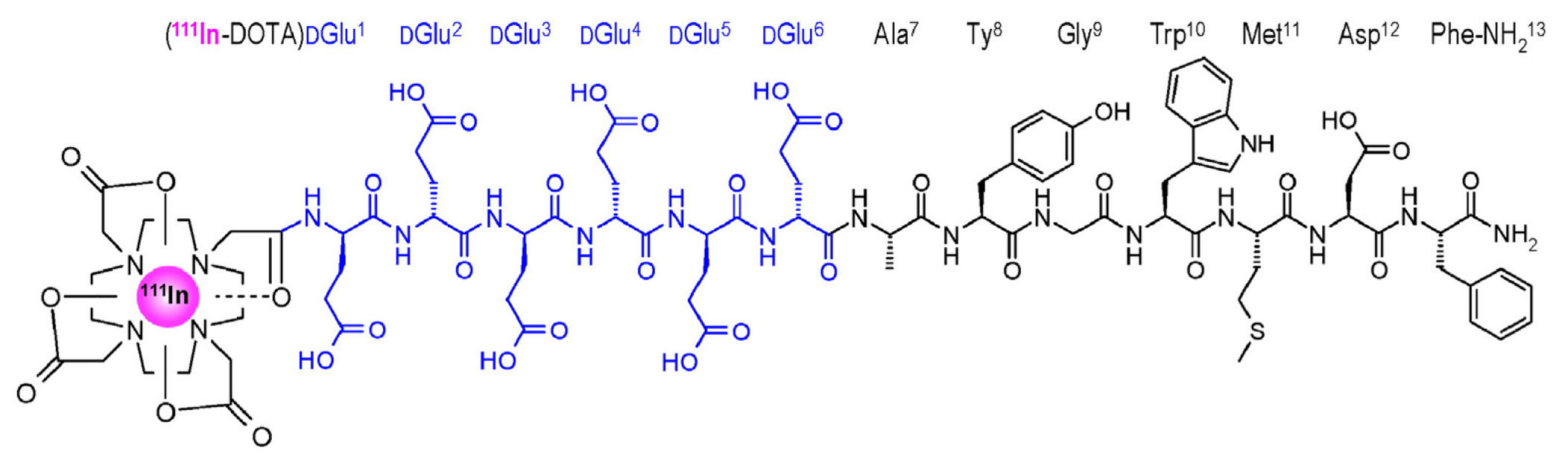
Chemical structure of ^111^In-CP04 with the DGlu^1–6^-chain highlighted in blue. (For interpretation of the references to colour in this figure legend, the reader is referred to the web version of this article.)

From a series of such analogues the linear peptide radioligand ^111^In-DOTA-(DGlu)_6_-Ala-Tyr-Gly-Trp-Met-Asp-Phe-NH_2_ (^111^In-CP04, also named PPF11; Fig. 1) was selected as the best candidate for a subsequent phase I clinical study in the diagnosis and therapy of human MTC, owing to its high tumour uptake and retention as well as the favorable tumour-to-kidney ratios attained (Aloj et al., 2011; Laverman et al., 2011; Ocak et al., 2011).

For conducting a multicenter clinical trial in Europe, an Investigational Medicinal Product Dossier (IMPD) has to be submitted and approved by the Authorities. Such IMPD is based on the common technical dossier (CTD) which for radiopharmaceuticals should additionally include information related to radioactivity and radiolabelling procedures (Todde et al., 2014). Data on the chemical and pharmaceutical development and characterization of a freeze-dried kit formulation for radiolabelling of the radiopharmaceutical precursor CP04 with ^111^In (chapter 2.1 of the CTD) has been recently presented (Pawlak et al., 2016). We herein further describe the additional data generated for translating this promising radiopharmaceutical into a first in human clinical trial. This includes the non-clinical pharmacology, pharmacokinetics and toxicity data. Initial preclinical data already have been reported (Aloj et al., 2011; Laverman et al., 2011; Ocak et al., 2011; Kolenc Peitl et al., 2015). In the present work we additionally report on the specific requirements for a radioactive medicinal product, including specific toxicity studies (Koziorowski et al., 2016), animal tumour models and estimation of the expected human radiation dose extrapolated from animal data, essential for the regulatory approval of the clinical trial.

## 2 Materials and methods

### 2.1 Chemicals and peptides

If not otherwise stated, chemicals, materials and solvents were of pharmaceutical grade for kit preparation and reagent grade for other experiments and were used without further purification. l-methionine was provided by SAFC chemicals (Cleveland OH), ascorbic acid and gentisic acid (Ultrapure quality) were provided by Sigma Aldrich (St. Louis, MO). The CP04-precursor in GMP grade was provided by PiChem (Graz, Austria) and CP04 freeze-dried kits for the preparation of ^111^In-CP04 were prepared as previously described (Pawlak et al., 2016).

### 2.2 Animals

For acute intravenous toxicity studies Crl:NMRI (SPF) female mice provided by Charles River Laboratories (Germany) of 7–9 weeks of age and a body weight of 26–33 g were used. For extended acute single dose toxicity study in rats, 45 female and 45 male Wistar Hannover RccHan™ rats were used purchased from Harlan Laboratories, B.V. (The Netherlands). Animals were 7–7 weeks of age and the body weights were 195–235 g for males and 164–199 g for females. For in vivo stability tests and biodistribution/dosimetry studies healthy male Swiss albino mice from NCSR “Demokritos” Animal House were used, of 8–10 weeks of age and a body weight of 20–25 g. For the CCK2R-expressing tumour model, male SCID mice from NCSR “Demokritos” Animal House were used, of 6 weeks of age on arrival day and a body weight of 20 ± 2 g.

### 2.3 Toxicity studies

#### 2.3.1 Acute toxicity of CP04 in mice

The CP04 precursor was dissolved in phosphate buffered saline (PBS pH 7.4) and was administered at a dose of 178.5 μg/kg body weight and a dose volume of 5 mL/kg body weight into the caudal vein of two groups of three mice (Acute Intravenous Toxicity Study in Mice, Harlan Laboratories Study #D04314). The test procedures were based on OECD Guideline 423 “Acute Oral Toxicity – Acute Toxic Class Method”, adopted on 17th December 2001. In brief, the animals were examined daily during the acclimatisation period. Mortality, viability and clinical signs were recorded. All animals were examined for clinical signs within the first 30 min and approximately 1, 2, 3 and 5 h after treatment on test day 1 and once daily during test days 2–15. Mortality/viability was recorded within the first 30 min and approximately 1, 2, 3 and 5 h after administration on test day 1 (with the clinical signs) and twice daily during days 2–15. Body weights were recorded on test day 1 (prior to administration) and on test days 8 and 15. All animals were necropsied and examined macroscopically.

#### 2.3.2 Extended acute single dose toxicity study in rats

The study was designed according to the microdosing concept as described in ICH guideline M3 (R2) (“Note for guidance on non-clinical safety studies for the conduct of human clinical trials and marketing authorization for pharmaceuticals” (CPMP/ICH/286/95)). In brief, 90 rats were distributed into three dose groups, each containing fifteen animals per sex (Table 1). The animals from Group 1 received only PBS, serving as controls, while groups II and III received the CP04 solution by single intravenous (bolus) injection at a low (89 μg/kg body weight: Group 2) and a high dose (890 μg/kg body weight: Group 3). A 24-h observation period followed (Allocation A), except for five animals per sex from each group for which treatment was followed by an additional 14-day treatmen*t*-free recovery period (Allocation B).

At day 2 blood samples were withdrawn from Allocation-A animals for haematology, clinical biochemistry and samples for urinary analyses were collected. These animals were necropsied on the same day and histological examinations were performed on an extended set of organs (adrenal glands, kidneys, liver, pancreas, sternum (including bone marrow), small intestine, stomach, thyroid/parathyroid glands and gross lesions). The animals from Allocation-B remained untreated for the following 14 days, but on day 15 underwent the same procedure described above (Extended Acute Intravenous (Bolus) Toxicity Study in Rats, Harlan Study #S47364).

### 2.4 Radiolabeling and quality control

For labelling, a sterile, non-pyrogenic solution of non-carrier-added ^111^InCl_3_ in 0.05 M HCl was supplied from Mallinckrodt Medical (Petten, NL). The solution contained 185 MBq (5 mCi) of ^111^InCl_3_ per 0.5 mL at time of calibration (specific activity of > 1.85 GBq/μg indium at time of calibration). CP04 (10 μg POLATOM kit, 4.88 nmol) was dissolved in 400 μL water for injection (Pharm.Eur., Fresenius Kabi, Halden, Norway), ^111^InCl_3_ was added (~120 μL, 94.35 MBq) and the mixture was incubated for 12 min at 85 °C. The “wet-labelling” of CP04 with ^111^In was completed in an Eppendorf vial. Freshly prepared sodium ascorbate buffer (10 mM) was added in the vial, followed by ^111^InCl_3_ solution (37–74 MBq), Met (1000 nmol) and CP04 (10 nmol). The mixture was left to react at 80 °C for 20 min. Before quality control tests, EDTA in 0.1 M acetate buffer (pH 4.6) was added to a final concentration of 1 mM to the labelling reaction mixture as a “free” ^111^In^3+^ scavenger. Quality control was conducted by HPLC analysis on (system 1) a RP-18 XTerra column (5 μm, 3.9 mm × 20 mm) eluted at a flow rate of 1 mL/min with the linear gradient: 100% A/0% B to 40% A/60% B within 60 min (A = 0.1% aqueous TFA and B = MeCN).

### 2.5 In vivo stability of ^111^In-CP04 in mice

A bolus of ^111^In-CP04 (100 μL, 11 MBq, ≈3 nmol peptide) was injected in the tail vein of healthy mice. Blood withdrawn 5 min post injection (pi) was directly placed in pre-chilled polypropylene tubes containing EDTA and Met on ice. Samples were centrifuged (10 min, 2000 ×*g*/4 °C, in a Hettich, Universal 320R, centrifuge, Tüttlingen, Germany), plasma was collected, mixed with chilled MeCN in a 1/1 v/v ratio and centrifuged again (10 min, 15,000 ×*g*/4 °C). Supernatants were concentrated to a small volume under a gentle N_2_-flux at 40 °C, diluted with physiological saline (≈400 μL) and filtered through a Millex GV filter (0.22 μm). Aliquots thereof were analysed by HPLC under the following conditions (system 2): A RP-18 Symmetry Shield column (5 μm, 3.9 mm × 20 mm) was eluted at a flow rate of 1 mL/min with the linear gradient: 100% A/0% B to 40% A/60% B within 60 min (A = 0.1% aqueous TFA and B = MeCN); the t_R_ of intact ^111^In-CP04 was determined by co-injection of a parent radioligand sample.

### 2.6 Pharmacokinetics of ^111^In-CP04 in healthy mice

A bolus of ^111^In-CP04 (100 μL, 111 kBq, 10 pmol peptide) was injected via the tail vein in healthy mice and animals were euthanized in groups of four at 30 min, 1 h, 4 h, 24 h, 48 h and 72 h pi. Samples of blood and tissues of interest were excised, weighted and counted for radioactivity in the gamma counter (automated well-type multi-sample gamma counter; NaI(Tl) 3″ crystal, Canberra Packard Auto-Gamma 5000 series instrument). Data was calculated as percent injected dose per gramtissue (%ID/g) with the aid of suitable standards of the injected dose.

### 2.7 Biodistribution of ^111^In-CP04 in mice bearing double A431-CCK2R(±) xenografts

The human epidermoid A431 cell line transfected to stably express the CCK2R (A431-CCK2R(+)) or devoid of CCK2R expression (A431-CCK2R(−)) used for tumour induction in this work was a gift from Prof. O. Boerman (Department of Nuclear Medicine, Radboud University Nijmegen Medical Centre, Nijmegen, The Netherlands) and Prof. L. Aloj (Istituto di Biostrutture e Bioimmagini, Consiglio Nazionale delle Ricerche, Naples, Italy) and cells were cultured as previously reported (Aloj et al., 2004). Suspensions of freshly harvested A431-CCK2R(±) cells (~150 μL, 1.6 × 10^7^/1.4 × 10^7^) in normal saline were subcutaneously injected in the flanks of mice. A week later well palpable tumours (260 ± 80 mg) developed at the inoculation sites and biodistribution was conducted.

At the day of the experiment, a bolus of ^111^In-CP04 (100 μL, 111 kBq, 10 pmol peptide) was injected in the tail vein of mice. Animals were euthanized in groups of four at 1 h, 4 h and 24 h pi; in a separate 4-h group animals received gelofusine (100 μL) together with the radioligand. A further 4-h group of mice was injected with ^111^In-CP04 prepared by “wet labelling” (100 μL, 111 kBq, 10 pmol peptide). Mice were dissected and samples of blood and tissues of interest as well as tumours were excised, weighted and counted for radioactivity in the gamma counter. Data was calculated as percent injected dose per gram tissue (%ID/g) with the aid of suitable standards of the injected dose and represent mean ± SD. In all above experiments, analysis of the solution used in the biodistribution experiment was conducted prior to and after completion of all animal injections.

Statistical analysis using the unpaired two-tailed Student’s t-test was performed to compare values between A431-CCK2R(+) and A431-CCK2R(−) tumours, as well as kidney, stomach and A431-CCK2R(+) tumour values between 4 h control and gelofusine-treated animal groups; values *P* < 0.05 were considered statistically significant (Table 2). The pharmacokinetic profile was determined by fitting an exponential curve through the organ time-activity data according to the least-squares method. The number of exponentials in the fitted curve was analysed according to the Aikake Information Criterion (AICc). All fitting was performed with Graphpad Prism version 5.

All animal experiments were approved by national authorities and were carried out in compliance with national and European guidelines.

### 2.8 Dosimetric calculations

Dosimetric calculations were based on biodistribution of ^111^In-CP04 in healthy mice from 30 min to 72 h pi (Table 2). Clearance kinetics in each organ was determined by exponential curve fitting. The time-integrated activity concentration (TIAC) was obtained by integration of this exponential curve folded with the decay curve of ^111^In and ^177^Lu.Mouse dosimetry was calculated for both ^177^Lu and ^111^In using the TIAC and the 25 g RADAR mouse absorbed dose rates per unit activity S for each source and target organ combination (Keenan et al., 2010; Konijnenberg et al., 2014).

Extrapolation of biodistribution uptake data in mice to humans was based on two options for interspecies scaling (Stabin, 2008).

1. $${\left(\frac{\%\text{IA}}{organ}\right)}_{human}=\left[{\left(\frac{\%\text{IA}}{g}\right)}_{mouse}\times M_{mouse}(kg)\right]\times {\left(\frac{m(g)}{M(kg)}\right)}_{human}$$

with *m* the organ mass and *M* the total body weight of mouse or human and with $\frac{\%\text{IA}}{g}\text{and}\frac{\%\text{IA}}{organ}$ the percentage of the injected activity concentration and per organ, respectively. The mouse organ TIACs are multiplied with the ratio of mouse and human body weights $\frac{M_{mouse}}{M_{human}}$ to obtain the extrapolated human TIAC, and this multiplied with the organ mass (from the MIRD standard man phantom) yields the residence time in each source organ.

2. $${\left(\frac{\%\text{IA}}{organ}\right)}_{human}={\left(\frac{\%\text{IA}}{organ}\right)}_{mouse}={\left(\frac{\%\text{IA}}{g}\right)}_{mouse}\times m_{mouse}$$

in this method the uptake per organ is extrapolated one-to-one from mouse to man. The extrapolated human residence time is obtained by multiplying the mouse TIAC or each source organ with its mouse organ mass.

The extrapolated human source organ residence times were used as input in the Olinda/EXM dosimetry software to calculate the absorbed doses per administered activity in humans (Stabin et al., 2005).

## 3 Results

### 3.1 Toxicology

#### 3.1.1 Acute toxicity of CP04 in mice

All animals survived until the end of the study with no systemic signs of toxicity evident throughout the entire observation period. The animal body weight was within the range commonly recorded for this strain and age. No macroscopic findings were recorded at necropsy. The median lethal dose (LD_50_) of CP04 after single intravenous administration to female mice observed over a period of 14 days was found to be ≥ 178.5 μg/kg body weight.

#### 3.1.2 Extended acute single dose toxicity of CP04 in rats

All animals survived the 24-h study period with no clinical signs being recorded. Under the experimental conditions, single bolus injection of CP04 in rats at a dose of 890 μg/kg body weight caused a moderate decrease in mean food consumption and body weight -gain and a marked decrease in the reticulocyte count in the male rats 14 days after administration. Based on pathological examination, the no-observed-adverse-effect-level (NOAEL) was initially established at 890 μg/kg body weight. However, the reversibility of the effect recorded in the reticulocyte count remained unknown and it was not possible to assess whether it represents a true adverse effect. Accordingly, the lower dose of 89 μg/kg body weight was eventually set as NOAEL, supported also by the absence of toxicological adverse alterations in morphology, functional capacity, growth, development or life-span of treated animals.

### 3.2 Metabolic stability of^111^In-CP04 in mice

**Fig. 2:**
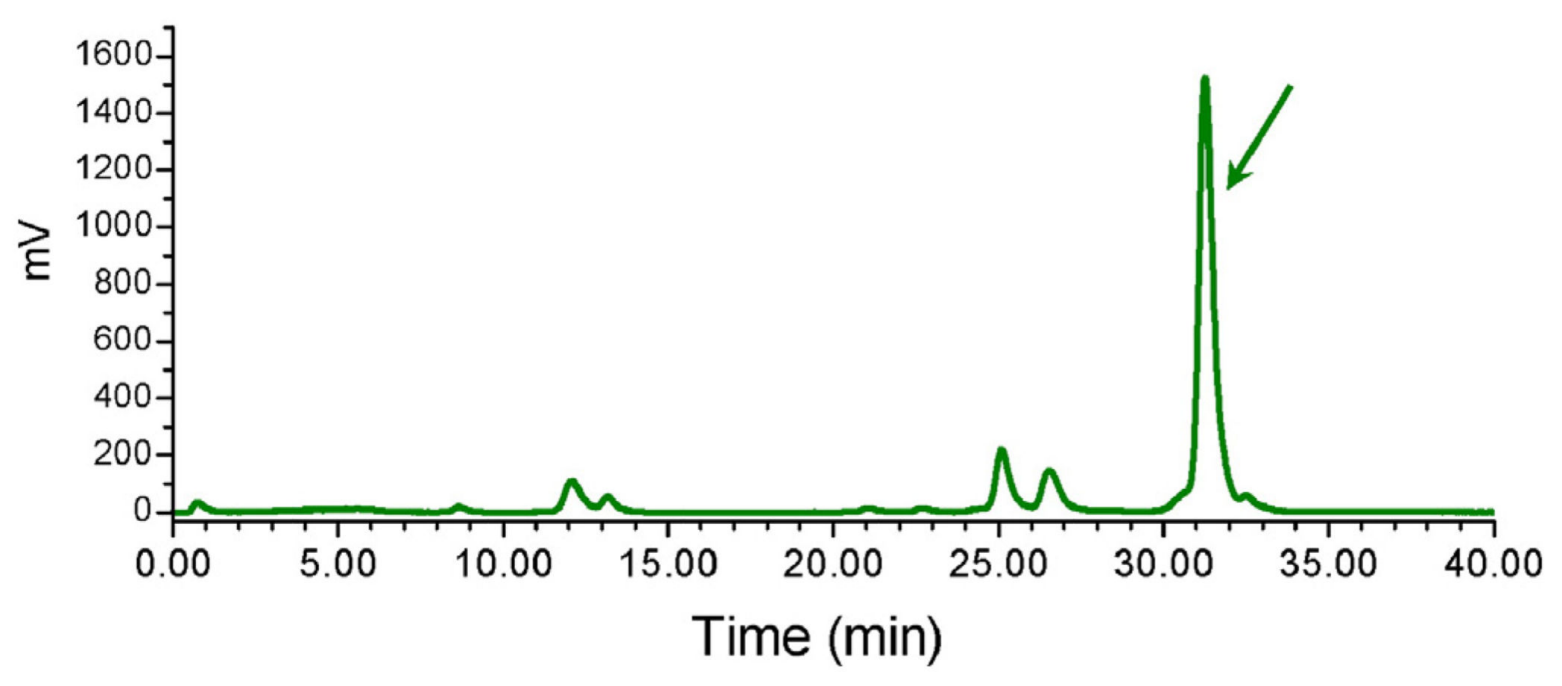
Typical HPLC (system 2) radiochromatogram of blood sample collected 5 min after intravenous injection of ^111^In-CP04 in Swiss albino mice with 70% intact radiotracer still detected; the t_R_ of intact ^111^In-CP04 is indicated by the arrow.

According to the HPLC analysis of mouse blood samples collected 5 min pi > 70% of ^111^In-CP04 remained intact in peripheral mouse blood. A radiochromatogram of a representative mouse blood sample is included in Fig. 2.

### 3.3 Biodistribution of^111^In-CP04 in healthy mice

**Fig. 3:**
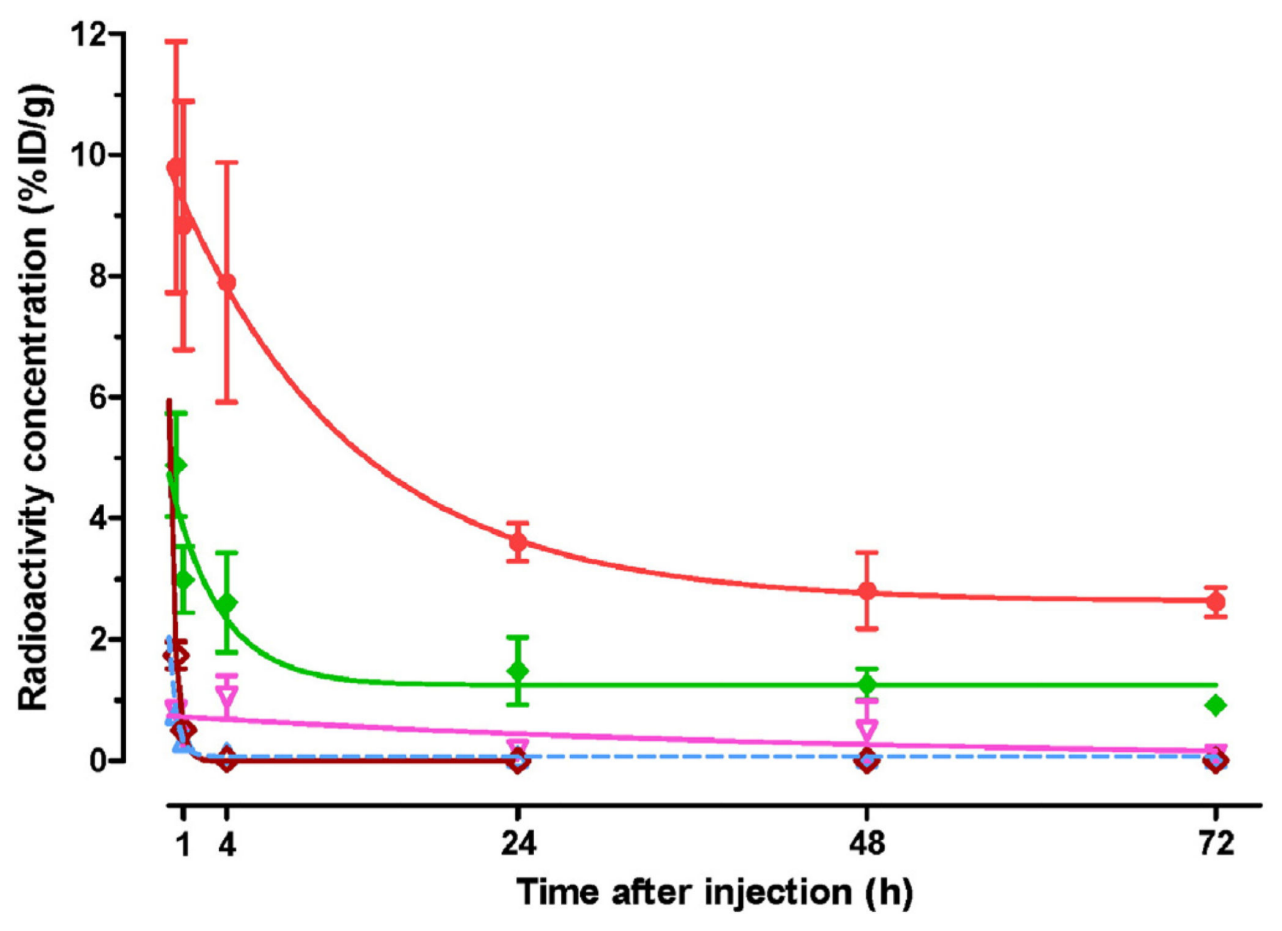
Time-dependent radioactivity clearance after injection of ^111^In-CP04 in Swiss albino mice shown for selected tissues; 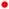 kidneys, 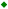 stomach, 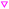 intestines, 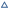 liver, 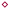 blood; values represent mean %ID/g ± SD, *n* = 4. Single-exponential fits through the data are shown by the curves with corresponding color. (For interpretation of the references to colour in this figure legend, the reader is referred to the web version of this article.)

Biodistribution data of ^111^In-CP04 in healthy mice at 30 min, 1, 4, 24, 48 and 72 h pi is summarized in Table 2 as %ID/g (mean ± SD, *n* = 4), whereas time-dependent clearance curves selectively for kidneys, stomach, liver, intestines and blood are included in Fig. 3. The radiotracer cleared very rapidly from the blood and the body of mice predominantly via the kidneys and the urinary tract. Radioactivity was retained mainly in the stomach (a CCK2R-positive organ) and in the kidneys.

### 3.4 Biodistribution in A431-CCK2R(±) tumour-bearing mice

**Fig. 4:**
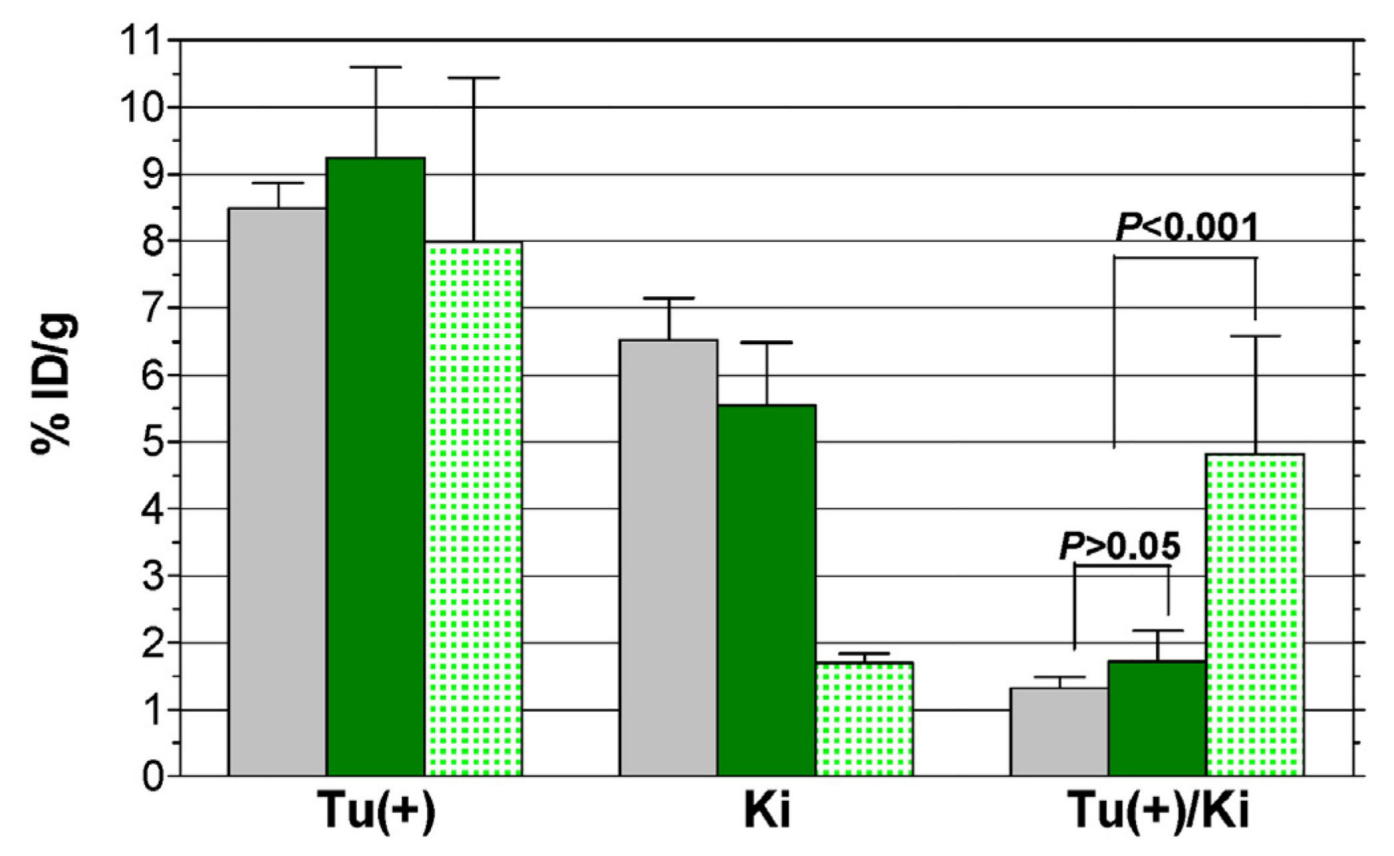
Biodistribution of ^111^In-CP04 (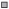) prepared by wet-labelling, (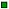) after kit-reconstitution and (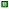) during gelofusine coinjection in A431-CCK2R(+) tumour bearing SCID mice at 4 h pi. Results expressed as mean %ID/g ± SD are selectively shown for comparison for Tu(+): tumour, Ki: kidneys and Tu(+)/Ki: tumour-to-kidney ratios. Statistical analyses were performed using the unpaired two-tailed Student’s *t*-test.

Biodistribution data of ^111^In-CP04 in mice bearing double A431-CCK2R(±) xenografts at 1, 4, and 24 h pi is presented in Table 3 as %ID/g (mean ± SD, *n* = 4). Additional animal groups at 4 h pi are also included in the Table 3, corresponding to mice coinjected with gelofusine or receiving ^111^In-CP04, prepared by wet-labelling. Selective data for A431-CCK2R(+) tumours, kidneys and kidney-to-blood ratios for 4-h animal groups is included in Fig. 4 for comparison. In agreement to findings of the healthy mice study, the radiotracer cleared very rapidly from the blood and the background via the kidneys and the urinary system, showing some retention in the CCK2R-possitive stomach and in the kidneys. It is interesting to note that gelofusine coinjection induced a significant (*P* < 0.001) reduction of renal uptake from 5.55 ± 0.94%ID/g to 1.69 ± 0.15%ID/g at 4 h pi, with the radioactivity excreted rapidly into urine. A CCK2R-independent process seems to be involved in the prolonged retention of the radiotracer in mouse kidney, since the CCK2R-positive stomach and tumour were not affected by gelofusine injection.

Another significant finding is the indistinguishable biodistribution profile between the ^111^In-CP04 prepared from the freeze-dried kit and from wet-labelling. No significant difference in the radioactivity levels in the A431-CCK2R(+) tumours was observed between kit and wet-labelling animal groups (9.24 ± 1.35%ID/g and 8.49 ± 0.39%ID/g, respectively; *P* > 0.05), or between untreated and gelofusine-treated mice (9.24 ± 1.35%ID/g and 7.99 ± 2.45%ID/g, respectively; *P* > 0.05). The high uptake of the radiotracer in the human xenografts expressing the CCK2R, but not in the tumours devoid of CCK2R-expression suggests in vivo CCK2R-specificity.

### 3.5 Dosimetric calculations

Total body clearance was calculated to be 90% with T_1/2_ = 17 min and 10% with T_1/2_ = 38 h, whereas a gastrointestinal tract clearance of 2.5% was observed. Mouse dosimetry was calculated for both ^111^In and ^177^Lu and is presented in Table 4. Based on mouse data and adopting two interspecies scaling models for extrapolating animal to human data the expected absorbed doses per injected activity in humans were calculated, leading to very comparable effective doses for both options of 0.045 and 0.044 mSv/MBq (Table 5). Assuming a radioactivity dose of 220 MBq ^111^In is injected in a patient, a whole body radiation dose of 9.9 mSv should be expected. This estimation needs to be eventually verified during the clinical study.

## 4 Discussion

The therapeutic options for metastatic MTC are quite limited. Whereas classical cytotoxic chemotherapies are often linked to life-threatening toxicity, many novel targeted therapies (such as TKIs), although less toxic, still show considerable side effects. As a result, these systemic treatments are justified only for patients at significant risk of morbidity or mortality due to progressive spread disease. The rational design of new effective and less toxic site-directed drugs exploits the overexpression of molecular targets in MTC to specifically deliver cytotoxic loads to tumour cells. For example, minigastrin-based radionuclide carriers may deliver cytotoxic radiation to MTC lesions via CCK2R-targets on tumour cells while at the same time sparing surrounding healthy tissue. To improve the efficacy and limit the side-effects of this approach, radiolabeled minigastrin analogues should show high CCK2R-affinity, good in vivo stability for sufficient delivery to tumour-associated targets, rapid blood and physiological organ clearance preferably through the kidneys into urine. All above parameters are considered essential to optimize tumour to background ratios and thereby the therapeutic index of radiolabeled minigastrin analogues.

According to reported results, CP04 and ^111/nat^In-CP04 showed high CCK2R-affinity with the respective IC_50_s found in the lower nM range (Aloj et al., 2011; Kolenc Peitl et al., 2015). The ^111^In-CP04 radiotracer previously shown to be very stable in serum (Kolenc Peitl et al., 2015) and in animal tissue homogenates (Ocak et al., 2011), turned out to be fairly stable in peripheral mouse blood in the present work as well (Fig. 2). During a previous comparative biodistribution study within the COST BM0607, ^111^In-CP04 exhibited high uptake and retention in A431-CCK2R(+) tumours in mice combined with low kidney retention (Laverman et al., 2011) and cleared very rapidly from blood and the background. In the present work identical overall pharmacokinetic profile of ^111^In-CP04 prepared from the freeze-dried kit (Pawlak et al., 2016) and by wet-labelling was obtained. This finding is a strong demonstration of the efficacy and suitability of the freeze-dried kit formulation for further use in the clinical trial. The kidney retention of ^111^In-CP04 prepared from kit reconstitution was found low in agreement with previous data. Nevertheless, we aimed to further decrease the kidney retention by co-injecting the plasma expander gelofusine reported to reduce renal uptake of minigastrin radioligands in rodents (Gotthardt et al., 2007; Kolenc Peitl et al., 2015). Evidently, this approach was successful in inducing a significant (*P* < 0.001) reduction of the renal uptake of ^111^In-CP04 in our animal model without negatively affecting the radiotracer uptake in the A431-CCK2R(+) xenografts, and led to significant improvement of the tumour-to-kidney ratios (Table 3, Fig. 4).

Previous experience from small-scale studies performed in MTC patients using radiolabelled minigastrin analogues at a peptide dose of 5–10 μg (Froberg et al., 2009) and from the routine pentagastrin test, undesirable effects were recorded due to ligand-induced CCK2R-activation. Adverse reactions included increase of serum calcitonin levels, increase of heart rate, flushes, mild nausea, paraesthesia in the hands and dizziness. These effects were mild and disappeared spontaneously shortly after CCK2R-ligand administration. Biosafety risks related to the pharmacological effects seem to be acceptable for the intended application under certain conditions, such as the administration of the IMP under strict surveillance of the responsible physician and close mesh monitoring plan.

Nevertheless, the toxicity of the CP04 precursor was extensively tested in two animal species, revealing the very low toxicity of CP04. The higher dose of 890 μg/kg tested during the extended acute intravenous toxicity study in rats (Harlan Study #S47364) caused a marked decrease in the reticulocyte count, but the reversibility of this effect remains unknown. Therefore, the lower tested dose 89 μg/kg was set as the NOAEL. It should be noted that the human equivalent dose (HED) calculated for this lower NOAEL value was 14.4 μg/kg (HED (μg/kg) = NOAEL × rat km/human km = 89 μg/kg × 6/37 = 14.4 μg/kg). By taking into account a safety factor of 10, the maximum recommended starting dose (MRSD) for a first-in-human clinical trial would be 1.4 μg/kg.

According to the clinical trial protocol (Eudra-CT No 2015–000,805-38) only adults with confirmed metastatic MTC will be included in the study. Subjects will be intravenously injected with either a lower peptide dose of 10 μg/patient or a higher peptide dose of 50 μg/patient. It should be noted that both doses are below the MRSD. The rationale for selecting the above two doses in the clinical protocol are related to restrictions of CP04 radiolabeling with 200–250 MBq ^111^In, biosafety considerations due to peptide-induced side-effects and future theranostic use of ^177^Lu-CP04 for PRRT. Specifically, first applications of ^111^In-CP04 in man will be performed with the lower peptide dose. Once the biosafety of this approach is clinically established, the higher peptide dose useful for translation to ^177^In-CP04 for PRRT will be applied.

On the other hand, radiation-associated risk assessment is based on dosimetric calculations derived from animal data. Based on biodistribution data of ^111^In-CP04 in healthy mice for a 30 min-72 h period pi dosimetric calculations revealed the highest absorbed doses for kidneys (Table 5). By employing two different dosimetric scaling options to translate mouse data to the human situation almost identical values were obtained. The effective activity dose predicted for ^111^In-CP04 in the clinical trial was below 10 mSv (9.9 mSv/220 MBq). Thus, the effective dose of ^111^In-CP04 for two hypothetical successive applications would in fact be lower than that of the licensed product OctreoScan® for just one similar application in receptor imaging with SPECT (effective dose of 26 mSv/220 MBq according to SPC). Considering the severity of metastatic MTC this is a justifiable risk for the patient. The comparison of calculated absorbed dose for kidneys would be 27.5 mGy/220 MBq for ^111^In-CP04, while the respective value for Octreoscan® is being estimated at 108.3 mGy/220 MBq.

## 5 Conclusion

Toxicity studies have indicated the biosafety of CP04 for single intravenous injection in human planned in the clinical trial. Radiation-associated risk assessment based on dosimetry calculations from animal data has revealed a justifiable risk for the patients. Furthermore, biodistribution experiments in tumour-bearing mice have not only confirmed indistinguishable in vivo behaviour for ^111^In-CP04 prepared by the freeze-dried kit or by wet-labelling, but also the high and CCK2R-specific tumour-targeting efficacy of ^111^In-CP04 as well as the role of gelofusine in significantly reducing renal retention. This data is in favour of prompt implementation of the freeze-dried kit formulation without or with gelofusine coinjection in a first multicentre clinical trial in MTC patients.

## Figures and Tables

**Table 1:** Number of wistar rats per group according to dose, sex and monitoring.

Groups	1* (control)	2	3
Treatment	Vehicle	CP04	CP04
Dose levels	0 μg/kg	89 μg/kg	890 μg/kg
Male A	10	10	10
Male B	5	5	5
Female A	10	10	10
Female B	5	5	5

A: animals for main study (Allocation A); B: animals for recovery (Allocation B); 1*: group 1 received vehicle only.

**Table 2:** Biodistribution of ^111^In-CP04 in healthy Swiss albino mice.

	%ID/g tissue ± SD (*n* = 4)					
	^111^In-CP04					
Organs	30 min	1h	4h	24 h	48 h	72 h
Blood	1.74 ± 0.22	0.51 ± 0.14	0.02 ± 0.01	0.00 ± 0.00	0.01 ± 0.01	0.01 ± 0.01
Liver	0.77 ± 0.11	0.32 ± 0.15	0.12 ± 0.02	0.06 ± 0.01	0.06 ± 0.00	0.05 ± 0.01
Heart	0.70 ± 0.10	0.21 ± 0.04	0.03 ± 0.01	0.02 ± 0.01	0.02 ± 0.01	0.03 ± 0.02
Kidneys	9.80 ± 2.07	8.84 ± 2.05	7.90 ± 1.98	3.61 ± 0.31	2.81 ± 0.63	2.62 ± 0.24
Stomach	4.88 ± 0.85	2.99 ± 0.55	2.61 ± 0.82	1.48 ± 0.55	1.26 ± 0.26	0.92 ± 0.04
Intestines	0.82 ± 0.14	0.39 ± 0.12	1.05 ± 0.35	0.18 ± 0.14	0.08 ± 0.02	0.09 ± 0.04
Spleen	0.36 ± 0.04	0.14 ± 0.04	0.05 ± 0.01	0.04 ± 0.01	0.03 ± 0.01	0.04 ± 0.01
Muscle	0.59 ± 0.10	0.18 ± 0.04	0.04 ± 0.02	0.02 ± 0.01	0.03 ± 0.02	0.03 ± 0.02
Lung	1.05 ± 0.10	0.40 ± 0.10	0.06 ± 0.01	0.03 ± 0.01	0.02 ± 0.01	0.03 ± 0.01
Femur	4.62 ± 1.24	2.62 ± 0.42	0.96 ± 0.18	0.53 ± 0.04	0.55 ± 0.11	0.49 ± 0.06
Pancreas	0.67 ± 0.08	0.26 ± 0.08	0.10 ± 0.01	0.05 ± 0.01	0.04 ± 0.01	0.06 ± 0.03

**Table 3:** Biodistribution of ^111^In-CP04 in SCID mice bearing double A431-CCK2R(±) xenografts.

	%ID/g tissue, mean ± sd (*n* = 4)				
	[^111^*In*]CP04				
Organs	1 h	4 h	4 h + gelo	4 h ref.	24 h
Blood	0.55 ± 0.17	0.02 ± 0.01	0.02 ± 0.01	0.04 ± 0.02	0.00 ± 0.00
Liver	0.38 ± 0.12	0.11 ± 0.01	0.17 ± 0.14	0.12 ± 0.02	0.01 ± 0.01
Heart	0.24 ± 0.08	0.02 ± 0.01	0.03 ± 0.01	0.03 ± 0.01	0.02 ± 0.00
Kidneys	5.53 ± 0.78	5.55 ± 0.94	1.69 ± 0.15***	6.52 ± 0.63^ns^	3.29 ± 0.56
Stomach	2.60 ± 0.48	2.09 ± 0.32	2.15 ± 0.24^ns^	1.63 ± 0.20	1.58 ± 0.15
Intestines	0.36 ± 0.13	0.22 ± 0.08	0.39 ± 0.13	0.29 ± 0.18	0.10 ± 0.04
Spleen	0.18 ± 0.04	0.04 ± 0.02	0.07 ± 0.01	0.06 ± 0.02	0.05 ± 0.01
Muscle	0.14 ± 0.05	0.03 ± 0.02	0.03 ± 0.01	0.02 ± 0.01	0.01 ± 0.01
Lung	0.47 ± 0.13	0.04 ± 0.01	0.05 ± 0.03	0.04 ± 0.01	0.03 ± 0.01
Femur	0.96 ± 0.46	0.30 ± 0.22	0.27 ± 0.02	0.39 ± 0.08	0.24 ± 0.09
Pancreas	0.23 ± 0.08	0.06 ± 0.01	0.07 ± 0.01	0.07 ± 0.01	0.05 ± 0.01
Tumour(+)	12.60 ± 1.73	9.24 ± 1.35	7.99 ± 2.45^ns^	8.49 ± 0.39^ns^	4.98 ± 0.56
Tumour(–)	1.07 ± 0.29+++	0.19 ± 0.05+++	0.22 ± 0.03+++	0.14 ± 0.02+++	0.10 ± 0.02+++

Statistical analysis was performed using the Student’s *t*-test with *P* values indicating very significant (***/+++*P* < 0.001) and not significant (ns, *P* > 0.05) difference (+) between Tumour(+) (A431-CCK2R(+)) and Tumour(–) (A431-CCK2R(–)) and (*) between the 4 h and 4 h + gelofusine-treated mice. The values in column 4 h-ref. correspond to a separate group of mice injected with ^111^In-CP04 obtained by “wet-labelling”; no significant differences were observed between this group and the 4-h group injected with ^111^In-CP04 prepared by kit reconstitution.

**Table 4:** Dosimetry of ^111^In-CP04 and ^177^Lu-CP04 in mice.

	Absorbed dose per injected activity (mGy/MBq)	
Organs	^111^In-CP04	^177^Lu-CP04
Kidneys	56	400
Stomach wall	15	86
Bone	9	51
Large intestine	7	36
Small intestine	7	36
Total body	4	19
Pancreas	5	16
Lungs	3	12
Spleen	3	9
Liver	3	7

**Table 5:** Expected absorbed doses of ^111^In-CP04 in humans based on two scaling models for translating mouse to human data.

	Absorbed dose per injected activity (mGy/MBq)	
Organs	Option 1	Option 2
LLI wall	0.078	0.075
Small intestine	0.026	0.024
Stomach wall	0.012	0.011
ULI wall	0.037	0.035
Heart wall	0.005	0.003
Kidneys	0.039	0.124
Liver	0.005	0.009
Lungs	0.004	0.003
Muscle	0.010	0.009
Pancreas	0.008	0.017
Red marrow	0.010	0.012
Osteogenic cells	0.015	0.034
Spleen	0.007	0.011
Urinary bladder wall	0.459	0.456
Total body	0.011	0.010
Effective dose (mSv/MBq)	0.045	0.044
